# 4-Isopropyl-*N*-phenyl­cyclo­hexa-1,3-diene-1-carboxamide

**DOI:** 10.1107/S1600536810034859

**Published:** 2010-09-04

**Authors:** Yan-qing Gao, Shi-bin Shang, Jian Li, Xu Xu, Xiao-ping Rao

**Affiliations:** a Institute of Chemical Industry of Forest Products, Chinese Academy of Forestry, Nanjing 210042, People’s Republic of China

## Abstract

In the crystal structure of the title compound, C_16_H_19_NO, mol­ecules are linked through a pair of N—H⋯O hydrogen bonds, forming chains along the *a* axis.

## Related literature

The title compound was obtained by reaction of dihydrocumic acid, obtained from nopinic acid through dehydration, and aniline. For the preparation and structure of nopinic acid, see: Ma *et al.* (2007 ▶); Gao *et al.* (2009 ▶). For the preparation of dihydro­cumic acid, see: Jin & Ha (2006 ▶) For oxidation of β-pinene, see: Winstein & Holness (1955 ▶).
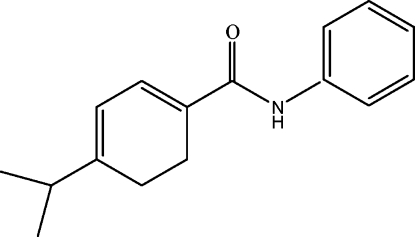


## Experimental

### 

#### Crystal data

C_16_H_19_NO
*M* *_r_* = 241.32
Triclinic, 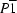

*a* = 5.226 (1) Å
*b* = 9.783 (2) Å
*c* = 13.810 (3) Å
α = 88.31 (3)°
β = 88.01 (3)°
γ = 76.13 (2)°
*V* = 684.9 (2) Å^3^
*Z* = 2
Mo *K*α radiation
μ = 0.07 mm^−1^
*T* = 293 K
0.30 × 0.20 × 0.20 mm

#### Data collection

Enraf–Nonius CAD-4 diffractometer
Absorption correction: ψ scan (North *et al.*, 1968 ▶) *T*_min_ = 0.979, *T*_max_ = 0.986
2789 measured reflections
2491 independent reflections
1901 reflections with *I* > 2σ(*I*)
*R*_int_ = 0.013
3 standard reflections every 200 reflections  intensity decay: 1%

#### Refinement

*R*[*F* ^2^ > 2σ(*F* ^2^)] = 0.056
*wR*(*F* ^2^) = 0.175
*S* = 1.01
2491 reflections
164 parameters
H-atom parameters constrained
Δρ_max_ = 0.24 e Å^−3^
Δρ_min_ = −0.22 e Å^−3^

### 

Data collection: *CAD-4 EXPRESS* (Enraf–Nonius, 1994 ▶); cell refinement: *CAD-4 EXPRESS*; data reduction: *XCAD4* (Harms & Wocadlo, 1995 ▶); program(s) used to solve structure: *SHELXS97* (Sheldrick, 2008 ▶); program(s) used to refine structure: *SHELXL97* (Sheldrick, 2008 ▶); molecular graphics: *SHELXTL* (Sheldrick, 2008 ▶); software used to prepare material for publication: *SHELXL97*.

## Supplementary Material

Crystal structure: contains datablocks I, global. DOI: 10.1107/S1600536810034859/ds2053sup1.cif

Structure factors: contains datablocks I. DOI: 10.1107/S1600536810034859/ds2053Isup2.hkl

Additional supplementary materials:  crystallographic information; 3D view; checkCIF report

## Figures and Tables

**Table 1:** Hydrogen-bond geometry (Å, °)

*D*—H⋯*A*	*D*—H	H⋯*A*	*D*⋯*A*	*D*—H⋯*A*
N—H0*A*⋯O^i^	0.86	2.27	3.054 (2)	151

Symmetry code: (i) 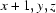.

**Fig. 1.**
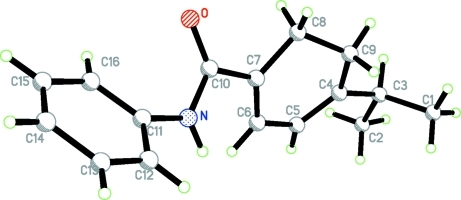
A view of the molecular structure of (I), showing displacement ellipsoids at the 30% probability level.

**Fig. 2.**
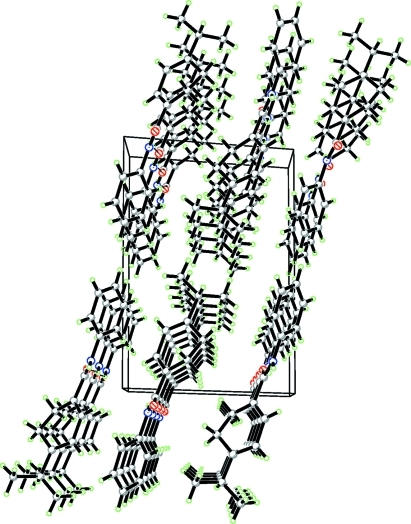
A view of the packing of the title compound.

## supplementary crystallographic information

### Comment

Nopinic acid is an important material prepared by oxidation of beta-pinene (Ma, 2007),and the crystal structure of nopinic acid has been reported (Gao,2009). From nopinic acid, dihydrocumic acid was obtained through dehydration. The title compound was got by reaction of dihydrocumic acid and aniline. In this work, we describe the crystal structure of the title compound. The asymmetric unit consists of one crystallographically independent molecule. The independent molecules are linked through a pair of N–H···O hydrogen bonds forming a polymer.

The molecular structure is shown in Fig. 1 and the crystal packing in Fig. 2, where the dash line indicates N–H···O hydrogen bonds. The bond lengths and angles are given in Table 1.

### Experimental

Dihydrocumic acid was (5.0 g) was dissolved in dichlomethane(100 ml) while stirring vigorously, thionyl chloride(6.6 ml) was dropped. The reaction was maintained during 6 h at the temperature of reflux. After removing dichlomethane and redundant thionyl chloride, the carboxylic acid chloride was obtained, which was then dropped in a mixture of dichlomethane(100 ml),triethylamine(6.1 ml) and aniline(5.6 g). The reaction was stayed over at room temperature. After reagent was romoved, the crude product was crystallized with ethanol, then the title conpound was gained. Crystals of the title compound suitable for X-ray diffraction were obtained by slow evaporation of a solution of ethanol. The crystal data were collected on an Enraf–Nonius CAD-4 difractometer. Data collection and cell refinement were performed using Enraf–Nonius *CAD-4 Software*.

### Refinement

All H atoms bonded to the C atoms were placed geometrically at the distances of 0.96–0.98 Å and included in the refinement in riding motion approximation with *U*_iso_(H) = 1.2 or 1.5*U*_eq_ of the carrier atom. H atoms bonded to the N atoms were fixed.

### Crystal data

**Table:**

C_16_H_19_NO	*Z* = 2
*M**_r_* = 241.32	*F*(000) = 260
Triclinic, *P*1	*D*_x_ = 1.170 Mg m^−^^3^
Hall symbol: -P 1	Mo *K*α radiation, λ = 0.71073 Å
*a* = 5.226 (1) Å	Cell parameters from 25 reflections
*b* = 9.783 (2) Å	θ = 9–13°
*c* = 13.810 (3) Å	µ = 0.07 mm^−^^1^
α = 88.31 (3)°	*T* = 293 K
β = 88.01 (3)°	Rod, colourless
γ = 76.13 (2)°	0.30 × 0.20 × 0.20 mm
*V* = 684.9 (2) Å^3^	

### Data collection

**Table:**

Enraf–Nonius CAD-4 diffractometer	1901 reflections with *I* > 2σ(*I*)
Radiation source: fine-focus sealed tube	*R*_int_ = 0.013
graphite	θ_max_ = 25.3°, θ_min_ = 1.5°
ω/2θ scans	*h* = 0→6
Absorption correction: ψ scan (North *et al.*, 1968)	*k* = −11→11
*T*_min_ = 0.979, *T*_max_ = 0.986	*l* = −16→16
2789 measured reflections	3 standard reflections every 200 reflections
2491 independent reflections	intensity decay: 1%

### Refinement

**Table:**

Refinement on *F*^2^	Secondary atom site location: difference Fourier map
Least-squares matrix: full	Hydrogen site location: inferred from neighbouring sites
*R*[*F*^2^ > 2σ(*F*^2^)] = 0.056	H-atom parameters constrained
*wR*(*F*^2^) = 0.175	*w* = 1/[σ^2^(*F*_o_^2^) + (0.1*P*)^2^ + 0.190*P*] where *P* = (*F*_o_^2^ + 2*F*_c_^2^)/3
*S* = 1.01	(Δ/σ)_max_ < 0.001
2491 reflections	Δρ_max_ = 0.24 e Å^−^^3^
164 parameters	Δρ_min_ = −0.22 e Å^−^^3^
0 restraints	Extinction correction: *SHELXL97* (Sheldrick, 2008), Fc^*^=kFc[1+0.001xFc^2^λ^3^/sin(2θ)]^-1/4^
Primary atom site location: structure-invariant direct methods	Extinction coefficient: 0.086 (14)

### Special details

**Table:**

Geometry. All e.s.d.'s (except the e.s.d. in the dihedral angle between two l.s. planes) are estimated using the full covariance matrix. The cell e.s.d.'s are taken into account individually in the estimation of e.s.d.'s in distances, angles and torsion angles; correlations between e.s.d.'s in cell parameters are only used when they are defined by crystal symmetry. An approximate (isotropic) treatment of cell e.s.d.'s is used for estimating e.s.d.'s involving l.s. planes.
Refinement. Refinement of *F*^2^ against ALL reflections. The weighted *R*-factor *wR* and goodness of fit *S* are based on *F*^2^, conventional *R*-factors *R* are based on *F*, with *F* set to zero for negative *F*^2^. The threshold expression of *F*^2^ > σ(*F*^2^) is used only for calculating *R*-factors(gt) *etc*. and is not relevant to the choice of reflections for refinement. *R*-factors based on *F*^2^ are statistically about twice as large as those based on *F*, and *R*- factors based on ALL data will be even larger.

### Fractional atomic coordinates and isotropic or equivalent isotropic displacement parameters (Å^2^)

**Table:**

	*x*	*y*	*z*	*U*_iso_*/*U*_eq_	
N	0.2449 (3)	0.16425 (18)	0.09778 (11)	0.0458 (4)	
H0A	0.3953	0.1648	0.0706	0.055*	
O	−0.1955 (3)	0.2062 (2)	0.07382 (11)	0.0737 (6)	
C1	0.3023 (9)	0.3380 (4)	−0.4181 (2)	0.1092 (13)	
H1A	0.2422	0.2525	−0.4160	0.164*	
H1B	0.4855	0.3171	−0.4026	0.164*	
H1C	0.2796	0.3800	−0.4818	0.164*	
C2	0.2353 (6)	0.5737 (3)	−0.3466 (2)	0.0852 (9)	
H2A	0.2377	0.6100	−0.4119	0.128*	
H2B	0.4093	0.5560	−0.3213	0.128*	
H2C	0.1162	0.6414	−0.3072	0.128*	
C3	0.1450 (5)	0.4384 (3)	−0.34559 (17)	0.0661 (7)	
H3A	−0.0370	0.4629	−0.3675	0.079*	
C4	0.1360 (4)	0.3777 (2)	−0.24416 (15)	0.0524 (6)	
C5	0.2943 (5)	0.2582 (2)	−0.21217 (15)	0.0574 (6)	
H5A	0.4178	0.2047	−0.2547	0.069*	
C6	0.2776 (4)	0.2095 (2)	−0.11182 (15)	0.0524 (5)	
H6A	0.4138	0.1391	−0.0872	0.063*	
C7	0.0686 (4)	0.2651 (2)	−0.05479 (14)	0.0459 (5)	
C8	−0.1475 (5)	0.3782 (3)	−0.09523 (18)	0.0720 (8)	
H8A	−0.2305	0.4400	−0.0436	0.086*	
H8B	−0.2800	0.3359	−0.1211	0.086*	
C9	−0.0495 (6)	0.4630 (3)	−0.17347 (19)	0.0803 (9)	
H9A	−0.1993	0.5186	−0.2078	0.096*	
H9B	0.0375	0.5277	−0.1439	0.096*	
C10	0.0261 (4)	0.2093 (2)	0.04320 (14)	0.0479 (5)	
C11	0.2431 (3)	0.1169 (2)	0.19526 (13)	0.0421 (5)	
C12	0.4082 (4)	0.1589 (2)	0.25855 (15)	0.0504 (5)	
H12A	0.5193	0.2149	0.2360	0.061*	
C13	0.4081 (5)	0.1179 (3)	0.35443 (16)	0.0610 (6)	
H13A	0.5183	0.1471	0.3966	0.073*	
C14	0.2471 (5)	0.0344 (3)	0.38850 (17)	0.0670 (7)	
H14A	0.2448	0.0082	0.4538	0.080*	
C15	0.0880 (5)	−0.0104 (3)	0.32473 (19)	0.0678 (7)	
H15A	−0.0184	−0.0691	0.3471	0.081*	
C16	0.0850 (4)	0.0304 (2)	0.22861 (16)	0.0552 (6)	
H16A	−0.0233	−0.0001	0.1863	0.066*	

### Atomic displacement parameters (Å^2^)

**Table:**

	*U*^11^	*U*^22^	*U*^33^	*U*^12^	*U*^13^	*U*^23^
N	0.0335 (8)	0.0635 (11)	0.0401 (9)	−0.0120 (7)	−0.0014 (7)	0.0048 (7)
O	0.0374 (8)	0.1279 (16)	0.0576 (10)	−0.0258 (9)	−0.0067 (7)	0.0244 (10)
C1	0.194 (4)	0.089 (2)	0.0460 (15)	−0.040 (2)	0.0213 (19)	0.0009 (14)
C2	0.098 (2)	0.0749 (18)	0.086 (2)	−0.0294 (16)	0.0137 (16)	0.0103 (15)
C3	0.0751 (16)	0.0754 (16)	0.0517 (13)	−0.0268 (13)	−0.0054 (11)	0.0128 (12)
C4	0.0595 (13)	0.0566 (13)	0.0453 (12)	−0.0216 (11)	−0.0079 (9)	0.0018 (9)
C5	0.0653 (14)	0.0595 (13)	0.0444 (12)	−0.0104 (11)	0.0073 (10)	−0.0029 (10)
C6	0.0548 (12)	0.0554 (12)	0.0449 (11)	−0.0095 (10)	−0.0012 (9)	0.0025 (9)
C7	0.0401 (10)	0.0592 (12)	0.0409 (11)	−0.0160 (9)	−0.0077 (8)	0.0010 (9)
C8	0.0486 (13)	0.101 (2)	0.0571 (14)	−0.0021 (13)	−0.0023 (10)	0.0125 (13)
C9	0.0794 (18)	0.0831 (19)	0.0611 (15)	0.0122 (15)	0.0004 (13)	0.0152 (13)
C10	0.0378 (11)	0.0647 (13)	0.0420 (11)	−0.0137 (9)	−0.0035 (8)	0.0012 (9)
C11	0.0331 (9)	0.0500 (11)	0.0400 (10)	−0.0036 (8)	−0.0006 (7)	0.0009 (8)
C12	0.0420 (11)	0.0614 (13)	0.0483 (12)	−0.0132 (9)	−0.0048 (9)	0.0024 (10)
C13	0.0540 (13)	0.0807 (16)	0.0450 (12)	−0.0084 (12)	−0.0103 (10)	−0.0001 (11)
C14	0.0555 (14)	0.0907 (18)	0.0456 (12)	−0.0021 (13)	0.0013 (10)	0.0171 (12)
C15	0.0517 (13)	0.0821 (17)	0.0687 (16)	−0.0180 (12)	0.0009 (11)	0.0259 (13)
C16	0.0451 (11)	0.0649 (14)	0.0581 (13)	−0.0180 (10)	−0.0079 (9)	0.0090 (11)

### Geometric parameters (Å, °)

**Table:**

N—C10	1.367 (2)	C6—H6A	0.9300
N—C11	1.411 (2)	C7—C10	1.474 (3)
N—H0A	0.8600	C7—C8	1.490 (3)
O—C10	1.226 (2)	C8—C9	1.494 (4)
C1—C3	1.502 (4)	C8—H8A	0.9700
C1—H1A	0.9600	C8—H8B	0.9700
C1—H1B	0.9600	C9—H9A	0.9700
C1—H1C	0.9600	C9—H9B	0.9700
C2—C3	1.507 (4)	C11—C16	1.377 (3)
C2—H2A	0.9600	C11—C12	1.386 (3)
C2—H2B	0.9600	C12—C13	1.372 (3)
C2—H2C	0.9600	C12—H12A	0.9300
C3—C4	1.509 (3)	C13—C14	1.370 (4)
C3—H3A	0.9800	C13—H13A	0.9300
C4—C5	1.333 (3)	C14—C15	1.381 (4)
C4—C9	1.478 (4)	C14—H14A	0.9300
C5—C6	1.458 (3)	C15—C16	1.374 (3)
C5—H5A	0.9300	C15—H15A	0.9300
C6—C7	1.338 (3)	C16—H16A	0.9300
			
C10—N—C11	125.04 (16)	C7—C8—C9	112.09 (19)
C10—N—H0A	117.5	C7—C8—H8A	109.2
C11—N—H0A	117.5	C9—C8—H8A	109.2
C3—C1—H1A	109.5	C7—C8—H8B	109.2
C3—C1—H1B	109.5	C9—C8—H8B	109.2
H1A—C1—H1B	109.5	H8A—C8—H8B	107.9
C3—C1—H1C	109.5	C4—C9—C8	114.0 (2)
H1A—C1—H1C	109.5	C4—C9—H9A	108.7
H1B—C1—H1C	109.5	C8—C9—H9A	108.7
C3—C2—H2A	109.5	C4—C9—H9B	108.7
C3—C2—H2B	109.5	C8—C9—H9B	108.7
H2A—C2—H2B	109.5	H9A—C9—H9B	107.6
C3—C2—H2C	109.5	O—C10—N	122.39 (18)
H2A—C2—H2C	109.5	O—C10—C7	121.11 (18)
H2B—C2—H2C	109.5	N—C10—C7	116.49 (16)
C1—C3—C2	110.9 (2)	C16—C11—C12	119.56 (18)
C1—C3—C4	114.6 (2)	C16—C11—N	122.18 (18)
C2—C3—C4	111.5 (2)	C12—C11—N	118.26 (17)
C1—C3—H3A	106.5	C13—C12—C11	120.2 (2)
C2—C3—H3A	106.5	C13—C12—H12A	119.9
C4—C3—H3A	106.5	C11—C12—H12A	119.9
C5—C4—C9	117.8 (2)	C14—C13—C12	120.5 (2)
C5—C4—C3	125.0 (2)	C14—C13—H13A	119.7
C9—C4—C3	117.1 (2)	C12—C13—H13A	119.7
C4—C5—C6	121.3 (2)	C13—C14—C15	119.2 (2)
C4—C5—H5A	119.3	C13—C14—H14A	120.4
C6—C5—H5A	119.3	C15—C14—H14A	120.4
C7—C6—C5	120.8 (2)	C16—C15—C14	120.9 (2)
C7—C6—H6A	119.6	C16—C15—H15A	119.6
C5—C6—H6A	119.6	C14—C15—H15A	119.6
C6—C7—C10	123.12 (19)	C15—C16—C11	119.6 (2)
C6—C7—C8	118.97 (19)	C15—C16—H16A	120.2
C10—C7—C8	117.54 (18)	C11—C16—H16A	120.2
			
C1—C3—C4—C5	14.4 (4)	C11—N—C10—C7	175.31 (18)
C2—C3—C4—C5	−112.5 (3)	C6—C7—C10—O	−142.6 (2)
C1—C3—C4—C9	−170.4 (3)	C8—C7—C10—O	30.4 (3)
C2—C3—C4—C9	62.7 (3)	C6—C7—C10—N	38.6 (3)
C9—C4—C5—C6	2.7 (3)	C8—C7—C10—N	−148.4 (2)
C3—C4—C5—C6	177.9 (2)	C10—N—C11—C16	41.7 (3)
C4—C5—C6—C7	14.9 (3)	C10—N—C11—C12	−138.7 (2)
C5—C6—C7—C10	172.7 (2)	C16—C11—C12—C13	−1.9 (3)
C5—C6—C7—C8	−0.2 (3)	N—C11—C12—C13	178.47 (19)
C6—C7—C8—C9	−29.0 (3)	C11—C12—C13—C14	0.5 (3)
C10—C7—C8—C9	157.8 (2)	C12—C13—C14—C15	1.2 (4)
C5—C4—C9—C8	−32.6 (3)	C13—C14—C15—C16	−1.6 (4)
C3—C4—C9—C8	151.8 (2)	C14—C15—C16—C11	0.2 (4)
C7—C8—C9—C4	44.7 (3)	C12—C11—C16—C15	1.5 (3)
C11—N—C10—O	−3.5 (3)	N—C11—C16—C15	−178.9 (2)

### Hydrogen-bond geometry (Å, °)

**Table:**

*D*—H···*A*	*D*—H	H···*A*	*D*···*A*	*D*—H···*A*
N—H0A···O^i^	0.86	2.27	3.054 (2)	151

Symmetry codes: (i) *x*+1, *y*, *z*.
